# Palliative Care Referral Patterns and Implications for Standardization in Cardiac ICU

**DOI:** 10.1007/s00246-024-03681-9

**Published:** 2024-10-22

**Authors:** Arshia Madni, Jocelyn Matheson, Amanda Linz, Austin Dalgo, Rumana Siddique, Anthony Merlocco

**Affiliations:** 1 University of Tennessee Health Sciences Center, Memphis, TN USA; 2 Division of Hospice and Palliative Medicine, LeBonheur Children’s Hospital, Memphis, TN USA; 3 The Heart Institute, Le Bonheur Children’s Hospital, Memphis, TN USA; 4 Children’s Foundation Research Institute, Le Bonheur Children’s Hospital, Memphis, TN USA

**Keywords:** CVICU, Pediatrics, Palliative care, End of life

## Abstract

Evidence suggests that pediatric palliative care involvement (PPC) is beneficial to medically complex patients. Historically, PPC involvement has been overlooked or delayed and varies by institution but PPC awareness has increased in cardiovascular ICUs (CVICU) and so we investigated frequency and timeliness of PPC referral for patients dying in ICU. Retrospective study of pediatric cardiac patients experiencing death in ICU to review PPC presence and timing of initial PPC, most recent PPC, and interventions, therapies, CPR, and presence of do-not-resuscitate DNR discussion. Fifty-four patients died during a 5-year period aged 11d–17y (54% male). PPC involvement occurred in 40/54 (74%). Of those patients without PPC, the Center to Advance Palliative Care (CAPC) guidelines would have supported PPC in 11/14 (79%). DNR discussion was more likely in PPC patients (63% vs 14%; *p* = 0.0011), though often only on DOD. Comparing *prior* to DOD, PPC patients were still more likely to have DNR discussion (55% vs 0%; *p* = 0.0003). PPC patients were no less likely to have CPR on DOD (28% vs 43%, *p* = 0.29). PPC occurred frequently in patients experiencing death in CVICU. However, frequently the initial PPC occurred within a week or day of death. Patients without PPC would often qualify under published guidelines. Standardization, timing, and patient identification for PPC will expand efficacy in CVICU.

## Introduction

The benefits of pediatric palliative care (PPC) extend to the family, the patient, and often members of the care team and include significant improvement in quality of life [1, 2]. The American Academy of Pediatrics and the Improving Palliative Care in the ICU Advisory Board and the Society of Critical Care Medicine have endorsed PPC as a best practice and key to comprehensive care, with attention to early involvement [3–8]. Additionally, guidelines have been provided by the Center to Advance Palliative Care for patient identification with disease and condition specificity for PPC [9].

However, despite these recommendations, involvement of the PPC team can be inconsistent among primary teams, particularly when standardized and automatic mechanisms are not in place, and this can differ among sub-specialty teams. While PPC involvement in conditions like bone marrow transplantation often occurs early, consistently, and improves outcomes for patients and families [10, 11], this is not always the case in other conditions. We observed that within our cardiovascular intensive care unit (CVICU), we lacked a formalized and standardized approach to PPC referral and this finding has been demonstrated previously elsewhere [12]. PPC plays an important role in having goals of care discussions, symptom management, attending to psychosocial distress of the patient and family while improving quality of life in pediatric patients with cardiac conditions particularly when faced with illness chronicity, ongoing hospitalizations, and frequent interventions especially following cardiovascular surgery [13–16]. Children with cardiac disease experience increasingly advanced care options and longer survival, while still suffering significant morbidity and mortality [17, 18]. Inconsistency and poor integration of PPC into CVICU care thus remain a pressing concern.

Pediatric cardiac patients experiencing PPC benefit from increased focus on goals of care and psychosocial support, especially towards goal-concordant care at end-of-life (EOL) [19]. A quaternary care center with experience coordinating PPC and intensive care has shown that PPC involvement resulted in less invasive procedures at EOL and decreased hospital charges [20]. Increased PPC awareness and integration into CVICU culture may aid in standardizing these practices nationally and beyond. We have found that anecdotal perceptions among our PPC providers and cardiologists regarding PPC involvement were varied and presumed low involvement rates. Given the increased awareness and publications over the last several years, we sought to study our own experience to better understand whether gaps exist in PPC referral, whether those lacking referral should have qualified under published CAPC guidelines, and how PPC timing related to care characteristics. Thus, we sought to study our own experience to better understand whether gaps exist in PPC referral, continuity, or timeliness, especially regarding the timing of the first PPC involvement.

## Methods

We conducted a retrospective single-institution cohort study in a tertiary pediatric institution. The institutional review board approved the study and waived the requirement for informed consent. CVICU patients who died between January 2018 and March 2023 were included, though some patients had been transferred to the neonatal (NICU) or pediatric intensive care units (PICU) prior to death. A CVICU database was interrogated for admissions and patient deaths among patients being followed by the cardiology team. Patients were included if they had cardiomyopathy, complex congenital heart disease (pre-or post-palliation), life-threatening arrhythmia, life-threatening coronary anomaly, or pulmonary hypertension. Eligibility for PPC involvement was assessed using CAPC guidelines [9]. Electronic medical records were reviewed for demographic data, presence of known genetic syndromes, hospital and CVICU length of stay, interventions utilized within 48 h of death including surgery or catheterization, requirement and frequency of extracorporeal membrane oxygenation (ECMO) and ventricular assist device (VAD), presence of a do-not-resuscitate (DNR) discussion, and CPR/code events. Timing of such events and interventions were analyzed, particularly those that happened on the day of death (DOD) or within 7 days of dying.

We reviewed the temporal relation with DOD, considering which proportion had their initial PPC involvement only in the 7 days proximal to their death or on the DOD itself. Of course some patients had several palliative care team visits after the initial PPC and in the days leading to death, so we reported it in both the “greater than” and “less than” 7-day groups to contextualize who received *initial* PPC on DOD.

We use PPC specifically for involvement of the subspecialty pediatric palliative care trained team, rather than any “palliative” focused discussion that often occurs in the ICU regarding end-of-life and withdrawal of life-sustaining therapies, but without PPC team input. In this manuscript our use of the term PPC involvement refers to any visit from the PPC team to the patient, family or care team.

PPC involvement was defined as *any* interaction with the palliative care consultation team, whether during the current hospitalization, a prior hospitalization, or outpatient (for whom there were none). Once a PPC was identified, it was considered an ongoing relationship (i.e. we considered “re-consultations” as visits from the team in patients already established).

Patients with PPC team involvement were subdivided into patients for whom their most recent PPC interaction occurred within 7 days of their death, and others for whom their most recent PPC interaction occurred more than 7 days prior to death. Next, we reviewed how many patients had their initial PPC involvement within 7 days of their death and if this initial involvement occurred on the day of the patient’s death itself. While this timeframe is somewhat arbitrary, we sought to explore PPC that was not so remote as to have lost input (such as patients for whom PPC occurred many months before death but not since), or those occurring so proximally as to limit therapeutic relationship building (such as those wherein the initial PPC involvement occurred within days of death or on the day of death). Given that the palliative needs of the patient generally increased closer to death, we determined based on our team communication, patient lists of “active” consultation, and provider service duration, 7 days would account for PPC involvement that was contributing most proximally to end-of-life needs.

Regarding DNR discussion, only presence or absence of discussion was reported due to inconsistencies in the charting.

We sought to describe the mode of death with respect to goals of care, clinical and prognostic changes, and withdrawal of life-sustaining therapy (WLST). In doing so, there was a broad spectrum of context to timing, clinical changes, and approaches to withdrawal. For instance, many patients experienced a cardiac arrest, were resuscitated to ECMO, and subsequently very soon after were disconnected from ECMO, whereas others were clinically “stable” on ECMO and after a goals of care (GC) discussion, experienced WLST. Thus, analysis in this realm was challenging and the spectrum of end-of-life experience and the consequent chart description was non-uniform. To wit, we encoded deaths into four general categories with consideration of prognostic changes, deterioration and timing, and WLST: 1. Re-evaluation of prognosis and changing goals of care resulting in WLST (GC-WLST), 2. Sudden and unanticipated deterioration and consequent WLST (SU-WLST), 3. sudden deterioration and the decision only to avoid escalation of care, dying due to the natural course such as bleeding or becoming asystolic (SU-NE), or 4. Sudden deterioration with active resuscitation efforts (SU-R). Some patients were resuscitated to ECMO but with severe neurologic injury or futile prognosis, and ECMO was withdrawn—because their status changed so significantly during the resuscitation we considered these patients to fall into the SU-R as we felt this was different from patients who had significant co-morbidities but without concerns for impending brain death such as fixed pupils.

Descriptive statistics were used to summarize demographic and clinical characteristics of patients with PPC involvement. Categorical variables were compared using chi-square tests or Fisher exact tests as appropriate. Continuous data was compared using the T- test and a p-value of less than 0.05 was considered statistically significant.

## Results

There were 54 deaths of cardiac patients occurring in the NICU, PICU, or CVICU during a 5-year period from January 2018 to March 2023. Overall, PPC involvement occurred in most patients who died (*n* = 40/54; 74%). Demographic characteristics and diagnoses are presented in Table 1. Of the 54 patients, nearly half were female (*n* = 25/54; 46%) and half were white (*n* = 24/54; 44%). Location of death included NICU (*n* = 6/54; 11%), PICU (*n* = 7/54; 13%) and CVICU (*n* = 41/54; 76%). At time of death, patients were aged 10 days to 17 years old (median 1.1 year). One patient in the PPC absent group had suffered TBI and non-accidental trauma and subsequently died shortly after presentation to the ER after cardiac arrest at home. This patient had single ventricle diagnosis and would have qualified for PPC according to the CAPC guidelines, otherwise all other patients experienced death related to their cardiac diagnoses.

**Table 1:** Demographic and diagnostic variables in patients with and without PPC

	Overall (*n* = 54)	PPC present (*n* = 40)	PPC absent (*n* = 14)	
Characteristic	Median (Range) or *n* (%)	Median (Range) or *n *(%)	Median (Range) or *n* (%)	*P* value
Age at death (years)	1.1 (10 days to 17 yrs)	0.62 (10 days to 17 yrs)	0.9 (19 days to 15 yrs)	0.22
Female sex	25 (46%)	19 (48%)	6 (43%)	0.76
Race				0.75
White	24 (44%)	17 (42%)	7 (50%)	
Black or African American	20 (37%)	16 (40%)	4 (29%)	
Hispanic	2 (4%)	1(3%)	1 (7%)	
Other	8 (15%)	6 (15%)	2 (14%)	
Diagnosis Category				0.15
1—Single ventricle cardiac anatomy s/p repair/palliation	24 (44%)	17 (43%)	7 (50%)	
2—Complete AV canal—biventricular	5 (9%)	5 (12%)	0 (0%)	
3—Biventricular dysfunction, arrhythmia, or obstructed LV filling*	18 (33%)	15 (38%)	4 (29%)	
4—Pulmonary hypertension	6 (11%)	3 (7%)	3 (21%)	
Genetic syndrome				0.12
Yes	21 (39%)	18 (45%)	3 (21%)	
Syndrome type				
Heterotaxy syndrome	7 (33%)	6 (33%)	1 (33%)	
Trisomy 21	6 (28%)	5 (28%)	1 (33%)	
Trisomy 18	1 (5%)	1 (6%)	0 (0%)	
Noonan syndrome	3 (14%)	3 (16%)	0 (0%)	
Jacobsen syndrome	1 (5%)	1 (6%)	0 (0%)	
DiGeorge syndrome	1 (5%)	0 (0%)	1 (33%)	
Duchenne/Becker Muscular Dystrophy	2 (10%)	2 (11%)	0 (0%)	

*Normal biventricular anatomy—cardiomyopathy or transplant failure, arrhythmia or coronary anomaly, or repaired, e.g., TOF, dTGA with complications or arrhythmia, or total anomalous pulmonary veins or cor triatriatum

We reviewed all deaths (54 patients) and the experience of those only with (40 patients) or without PPC (14 patients), thus the denominator noted will reflect the population for whom this was relevant. PPC referral presence or absence was not associated with demographic variables, or cardiac diagnosis. Of 14 patients without PPC, referral criteria would have supported involvement in 11/14 (79%) cases.

Table 2 presents clinical characteristics and PPC presence. Most patients with PPC had involvement within 7 days prior to DOD (*n* = 34; 63%), including initial or ongoing involvement, with 20 patients who had PPC involvement on the DOD (*n* = 20; 37%). PPC patients had a longer length of hospital stay (median 46.7 days vs 7.1 days; *p* = 0.049). PPC patients were also more likely to have had a DNR discussion (25; 63% vs 2; 14%—*p* = 0.0011) but pertinently 3 (8%) PPC patients and 2 (14%) PPC-absent patients had their first DNR discussion on the DOD, generally performed by the intensivist team. Thus, reviewing only DNR discussion prior to DOD, 22 (55%) PPC versus 0 PPC-absent patients experienced DNR discussion (*p* = 0.0003). PPC involvement did not predict CPR performance within 24 h of death (*p* = 0.29).

**Table 2:** Clinical characteristics of patients with and without PPC

	Overall	PPC present	PPC absent	*P* value
	(*n* = 54)	(*n* = 40)	(*n* = 14)	
	Median (Range) or *n* (%)	Median (Range) or *n* (%)	Median (Range) or *n* (%)	
Timing of first PPC to death (days)		30.5 (0–1723)		
Timing of most recent PPC to death (days)		0.5 (0–1009)		
CAPC guideline eligible		40 (100%)	11 (79%)	
Hospital length of stay	0.3 (34–681)	46.7 (1–681)	7.1 (0.3–104)	**0.0493**
CVICU contiguous days during most recent ICU stay	16.5 (0–169)	15 (0–169)	4 (0.3–104)	0.08
CPR within 24 h of death	17 (31%)	11 (28%)	6 (43%)	0.29
Mechanical ventilation	46 (85%)	35 (88%)	11 (79%)	0.42
ECMO/VAD	25 (46%)	20 (50%)	5 (36%)	0.36
Inotrope infusion	46 (85%)	35 (88%)	11 (79%)	0.42
Cardiac catheterization within 48 h	10 (19%)	6 (15%)	4 (29%)	0.26
Surgical intervention within 24 h	17 (31%)	11 (28%)	6 (43%)	0.29
DNR discussion				0.0011*
DNR discussion prior to DOD	22 (41%)	22 (55%)	0 (0%)	**0.0003**
Never	32 (59%)	18 (45%)	14 (100%)	
Initial DNR discussion on DOD	5 (9%)	3 (8%)	2 (14%)	0.45
Location of death				0.19
CVICU	41 (76%)	28 (70%)	13 (93%)	
PICU	7 (13%)	6 (15%)	1 (7%)	
NICU	6 (11%)	6 (15%)	0	
Mode of death				0.24^˄^
GC—WLST	22 (41%)	18 (45%)	4 (29%)	
SU—WLST	7 (13%)	5 (13%)	2 (14%)	
SU—NE	5 (9%)	4 (10%)	1 (7%)	
SU—R	20 (37%)	13 (32%)	7 (50%)	

*p-value of 0.0011 represents comparison of any DNR discussion including that on DOD; *p*-value of 0.0003 for DNR discussions occurring prior to the DOD versus none at all

GC-WLST = changing goals of care resulting in withdrawal of life-sustaining therapy, SU-WLST = sudden and unanticipated deterioration and consequent WLST, SU-NE = sudden deterioration, death due to lack of escalation of care only, SU-R = sudden deterioration with active resuscitation efforts but resulting in death

^˄^Due to a small patient population, those without active resuscitation (GC-WLST + SU-WLST + SU–NE) were compared to those with active resuscitation (SU-R)

In reviewing the mode of death of the patients, we initially considered comparing all groups, however due to a small patient population, we evaluated those dying without active resuscitation (GC−WLST + SU−WLST + SU−NE) compared with those dying during active resuscitation (SU-R) and found no relationship with presence of absence of PPC (*p* = 0.24). There was no significant correlation considering presence or absence of resuscitation and DNR discussion (*p* = 0.09), race and ethnicity (*p* = 0.34), or location of death (*p* = 0.54).

**Fig. 1:**
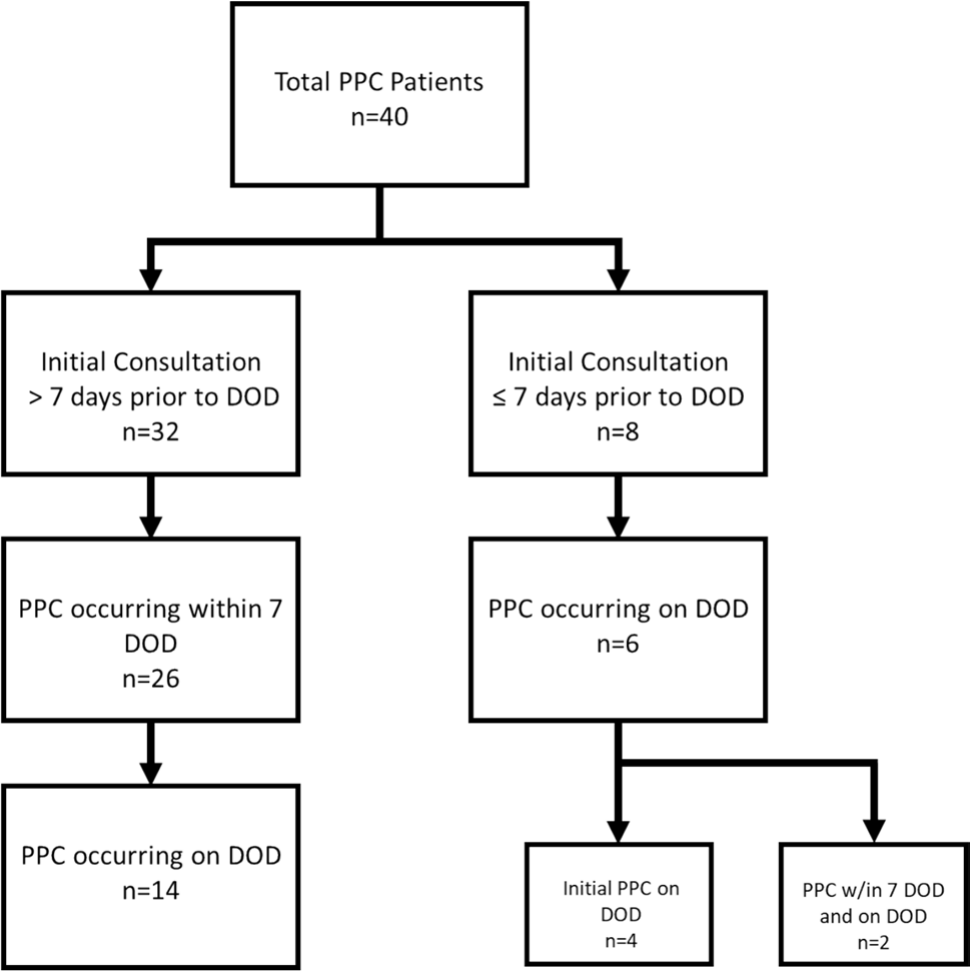
PPC involvement timing

We also reviewed any effects on the timing of PPC involvement, specifically whether PPC involvement occurred within 7 days prior to death. Figure 1 demonstrates how PPC timing related to DOD with review of whether the initial PPC involvement occurred prior to 7 DOD and subsequently how near DOD the initial or ongoing PPC involvement occurred. Of PPC patients, 8 (20%) patients had their initial involvement within 7 days of death, and of these, 6 patients experienced some PPC on DOD itself, including 4 (10%) patients whose *initial* PPC only occurred on DOD. The remaining 2 patients had PPC on DOD but it was not their initial PPC interaction.

Table 3 compares patients who had their most recent PPC involvement more than or less than 7 days prior to DOD. Most PPC patients had PPC involvement in the 7 days prior to DOD (34/40; 85%). Of all 40 patients with PPC, 20 (50%) had a PPC interaction on DOD itself. Three patients had very remote PPC involvement and PPC appears not to have been re-consulted during subsequent hospitalizations, with most recent PPC interaction to death of 147, 153, and 1009 days prior to DOD.

**Table 3:** PPC timing and characteristics

	Time from most recent PPC involvement to death		
	Greater than 7 days (*n* = 6)	Within 7 days (*n* = 34)	
	Median (Range) or *n* (%)	Median (Range) or *n* (%)	*p*-value
Timing of initial PPC to death	187.5 (66–1723 d)	24 (0–626)	0.19
Timing of recent PPC to death	90 (10–1009)	0 (0–7)	0.21
Hospital length of stay	53.4 (1–681)	46.7 (1.37–520.39)	0.52
CPR within 24 h of death	2 (33.3%)	9 (26.5%)	0.73
DNR discussion prior to DOD	5 (83.3%)	17 (50%)	0.13
Initial DNR discussion on DOD	1 (16.7%)	2 (5.9%)	0.36
Mechanical ventilation	6 (100%)	29 (85%)	0.32
ECMO/VAD	3 (50%)	17 (50%)	1.00
Inotrope infusion	6 (100%)	29 (85%)	0.32
Cardiac catheterization within 48 h	0 (0%)	6 (17.7%)	0.26
Surgical intervention within 24 h	2 (33.3%)	9 (26.5%)	0.73

One patient in the no PPC group was transferred from another institution for heart transplantation evaluation; however, this patient was deemed not a transplant candidate and subsequently deteriorated within 24 h. Thus, the patient was deemed not to have qualified for PPC based on the limited data. There was no documentation of PPC involvement at the prior institution.

## Discussion

Children experiencing congenital heart disease and pediatric cardiac conditions are living longer than at any point in history, but they also now experience increased chronic illness, frequent and prolonged hospitalizations, and repeated interventions secondary to cardiovascular surgeries. The addition of a palliative care team for this patient population can improve psychosocial outcomes, promote shared decision-making, assist with advanced care planning, and optimize symptom management [21, 22]. A recent scientific statement from the American Heart Association underscores the importance of an evidence-based approach to the awareness and delivery of palliative care for children with heart disease [23].

Our study was not designed to capture all patients who would benefit from PPC but rather just those who died in the CVICU. This was partially due to the nature of our database and also reflected the interests of our clinicians, who were experiencing distress at the uncertainty of how consistent PPC integration was for those who died, and how much end-of-life care involved PPC specialists.

At our institution it was unknown how consistently cardiac patients who qualify for CAPC-recommended PPC involvement were receiving this care and whether intensivist teams were referring patients. Previous data demonstrated low referral rates albeit in a cohort of all CVICU patients (rather than only those experiencing death) [12]. However, that data since publication of that data, PPC awareness has increased. Thus, our hope was that increased PPC awareness may manifest as high referral rates at our institution. Our data shows overall a high percentage of PPC referral rates in our CVICU. Unsurprisingly, we also found that PPC involvement tended to occur proximate to patient death, particularly within 7 days of death. We also found, though, that an important proportion of patients did not have PPC involvement prior to this timeframe, specifically 20% (8/40) had their *initial* PPC involvement within 7 days of death and that 10% (4/40) only had PPC involvement on the day they died. PPC is most efficacious when initiated early and so these findings help us identify an important issue upon which we can intervene. There are patients for whom involvement occurred but without any PPC interaction in the week of their death, specifically 15% (6/40) PPC patients had no involvement within 7 days of their death and while this may not indicate ineffective PPC, it may reflect communication issues among the teams, family hesitation due to biases and misconceptions about palliative care, or unanticipated changes in clinical course.

If PPC is requested initially on the week or day prior to death (which cannot always be anticipated), it may be challenging for PPC providers to fully connect with the family and team and to provide guidance on initial visits. PPC providers at our institution felt there were several barriers to earlier PPC involvement, generally due to diagnostic or presentation delay or decreased communication among teams. Thus, for some patients, *any* PPC involvement may represent a success of eventual PPC candidate identification and coordination. Interestingly there were no discrepancies in race or ethnicity when it came to PPC involvement.

While we found that PPC involvement occurred in most patients, a significant proportion had their initial involvement only in the 7 days proximal to their death or on the DOD itself. It is difficult to quantify any quality effects of these timeframe differences, but PPC involvement should occur early and often. Our study of the PPC timing demonstrated that in both those with initial PPC prior to and less than 7 days before death, most patients also had PPC involvement on DOD. We do not argue that DOD involvement itself is a marker of quality but have reported it in both the “greater than” and “less than” 7-day groups to contextualize who received *initial* PPC on DOD. The finding that 8 patients had initial PPC within 7 days of death (4 of which were only on the DOD) may demonstrate a failure to involve PPC early or optimally.

The delayed addition of palliative care to the patient care team is likely multi-factorial. Multiple stakeholders including cardiologists, intensivists, and surgeons may remain focused on previously discussed goals of *cure* even in the face of prognostication challenges, or due to limited review of these goals of care. However, studies have shown that the primary team often does recognize the importance of goals of care conversations [24]. Additional studies show that cardiologists see value in having the input of a specialty palliative team to discuss symptoms, quality of life and advanced care planning [25]. The findings from these studies support early integration of PPC, even when prognosis is unclear [26]. One important component of understanding how to reconcile these findings is better study of how PPC relates to benchmarks and quality metrics in the CVICU. Parental autonomy can be limited in this setting as well, further shifting the initiation to the primary team. We did not study the attitudes of our providers but were encouraged by the high referral rates we found. Providers at our institution do not commonly speak of the CAPC published guidelines on PPC referral and we still feel that these may be unfamiliar as a formal list. We did find that most (79%) of those without PPC involvement would have qualified and so education and familiarization with the CAPC guidelines may help decrease and standardize our approach.

Another barrier to early integration is the misconception that palliative care is primarily associated with end-of-life care by the medical team as well as caregivers [25]. Although we did not specifically investigate barriers to PPC involvement in our study, this has been noted anecdotally as a barrier at our institution.

An important discussion point and limitation in our study requires attention. DNR discussion was not found in the absent PPC patients and this finding is likely due to a combination of this discussion never occurring, difficult documentation due to no PPC involvement, or occurring only after the patient was assessed as experiencing futile care. Of course the latter situation accounts for many DNR discussions generally and requires further clarification. In our study we sought to show when DNR discussions occurred in ill patients prior to considering further support completely futile. In some absent PPC cases, there was notation in the EMR that after discussion with the family, care was withdrawn. Thus there is a discrepancy when considering CPR on DOD—some absent PPC patients had no CPR and no DNR discussion. However, we did not consider solely the decision to withdraw care to constitute a DNR discussion. While it certainly could be argued this qualifies, we felt the benefit of a DNR discussion lay in *planning*, not solely in *reacting*. Unfortunately, our EMR limited its documentation of the detail of these discussions. For instance, for one of the absent PPC patients, the EMR states only on DOD, “This 3-month-old boy with complex congenital heart disease arrested. The etiology most likely related to a respiratory event. The resuscitating team are unable to establish a perfusing rhythm. As a result, extracorporeal cardiopulmonary resuscitation is warranted. His physical exam was consistent severe hypoxic brain injury, so neurology service was consulted and recommended doing brain death exam. His EEG and exam were consistent with brain death. After discussing with family care was withdrawn and baby was expired.”

Our study focused on inpatient care, PPC involvement, and death. Regarding the mode of death results, understanding these findings is challenging because much of the resuscitation efforts were valid and warranted, thus we do not necessarily conclude that resuscitation occurred improperly, especially since PPC was involved. Rather, this spectrum may represent the tenuous and fragile nature of some patients who are at high risk of arrest and thus should have frequent review of prognosis, likelihood of success, goals of care, and end-of-life planning, even when resuscitation efforts are planned or expected.

Importantly, in several patients experiencing goals of care reevaluation and WLST, the PPC team was not directly involved in both PPC patients (one patient had a PPC visit 3 days prior but at time of WLST decision, only the primary care team was involved), and in those patients without any PPC involvement, indicating that intensivists are comfortable pursing these discussions without PPC input but highlighting opportunities to request consultation that did not occur.

Patients without PPC but who qualified for PPC generally experienced withdrawal of life-sustaining support in reaction to an assessment of futility, as described above. Whether this is meaningfully different than those experiencing withdrawal of life-sustaining support after a PPC discussion of DNR could be argued. Those without PPC and who would not have qualified for PPC (3 patients) died as follows: 1. collapsed at football, experienced cardiac arrest and subsequently developed cardiogenic shock in the ER and was transferred to CVICU, 2. after withdrawal of ECMO and decompensation due to worsening limb necrosis and renal dysfunction, and 3. after transfer for transplant evaluation experienced cardiac arrest and CPR with patient’s mother requesting compressions stop after 40 min.

Given the limitations in concluding how meaningful the difference in DNR discussion may have been, due to the limitations in EMR and bedside discussions that can occur with the intensivist, we focus on how PPC was experienced. While the DNR discussion difference was statistically different, our focus is less about delivering PPC specifically to increase DNR discussion, but rather that PPC involvement, while occurring frequently, could be utilized earlier, more consistently, and in a more standardized fashion.

Pediatric cardiology studies (specifically those with single ventricle physiology) show that early integration and continuity with the PPC resulted in meaningful and supportive therapeutic relationships between parents and care teams that were beneficial during highly stressful events [25]. Parents of children with cancer have shared they prefer their primary team to have goals of care conversations with them earlier rather than later in their child’s treatment course and are reticent to having these difficult conversations with teams they are not familiar with [10, 27]. With these data in mind, PPC involvement should become standardized and systemic at our institution and others. Our finding of three outliers highlights another gap in involvement that would benefit from standardization—3 patients who had initially been seen by PPC much earlier in their disease course (indicating that they had been correctly identified at some point in their disease course) were not seen again, indicating a likely failure by the primary team to contact the palliative care team later in their course. Thus, our data reveals a longer range of PPC timing (0-1723 days) than hospital length of stay (1–681 days) because the outlier patients had been discharged and readmitted without identification that they were PPC patients. Regarding the LOS, since this was significantly associated with PPC involvement one may expect that greater LOS resulted in PPC involvement and fewer interventions near end-of-life. However, in children who died, interventions within 48 h of death did not differ between PPC and no PPC groups. This may indicate that PPC involvement likely centered on the goals of care and communication facilitation while the primary team still focused on life-prolonging measures thus demonstrating no difference in CPR, catheterization, or surgery. Generally within 48 h of death it was not uncommon to continue optimizing medical invention with reconsideration on day of death.

While our CVICU team does not have formal CAPC evaluation there is consistent communication between the CVICU and palliative team, which may explain our high referral rates. Translating this congenial relationship into a model where palliative care champions exist within the CVICU setting may make consultations more fluid and earlier in the patient’s course.

Recently a PPC champion-based approach to increased integration has been developed by one institution’s CVICU which has resulted in improving education interventions, integrating PPC principles into patient care earlier, and increased mutual understanding between teams through an interdisciplinary focus [28]. This approach would be especially valuable when patients are re-admitted after the initial PPC consultation, which often only occurs in the inpatient setting at our hospital system. Palliative care integration in the ICU setting is soon to be a quality indicator for ICUs [29]. As consultations are requested based on provider decision-making, they can be limited by awareness of CAPC guidelines and as some studies have suggested, a degree of gatekeeping by the primary team. Certainly, there can be a stigma associated with palliative care to include “end-of-life care” which may delay referral, and other reservations about initiating referral to avoid reducing hope for patients and families. A model of timely palliative referral considers four elements: establishment of institution-specific criteria, system triggers for referral, routine screening for supportive care needs, and availability of outpatient palliative care resources [30]. This model is cited as being used for oncology care but can be applied appropriately to cardiac patients as well. To that end we propose that concerted efforts be made in order to integrate PPC early enough to improve outcomes for CVICU patients. Opportunities for improvement are multifaceted and can be broached through three principal areas related to: education, communication, and system processes.

1. *Communication* High quality communication between the CVICU team members and palliative team members can result in increased involvement as noted in our study. In addition, having a palliative care champion within the CVICU setting can result in more regular and consistent referrals earlier in the patient’s course and greater consensus-building and interdisciplinary cooperation [28].

2. *Education* Longitudinal education on the benefits of palliative care has led to increased palliative care consultations. One study in the NICU demonstrated that weekly case-based discussions including neonatology providers (physicians and advanced practice providers) and palliative care improved perceptions, knowledge, and comfort in palliative care. This led to increased confidence in palliative care which, in turn, resulted in increased overall PPC consultations [31].

3. *Systemic changes* Prognostication in cardiology patients can be challenging. Cardiologists and CVICU clinicians often focus the goals of care on long-term survival through significant interventions but should also include PPC early in the course given not only the longitudinal care PPC can provide but also the likelihood of care-goal changes. PPC may be integrated as part of an admission checklist visible to all ICU personnel including nursing staff, nurse practitioners, social workers, and medical residents on the service. A recent study utilized high visibility checklist and eligibility criteria with laminated cards for all ICU personnel, which instructed a palliative care consult and order for patients meeting criteria, circumventing the approval of the supervising physician on call. Though this criterion was initiated for oncology patients, the automated PPC involvement nearly doubled PPC consultations, significantly decreasing 30-day admission rates [32].

## Limitations

There are certainly limitations to this study. As this is a single-center study, generalizability may be limited. As such, there are no automatic PPC referral triggers as there may be in other centers, some of which have more well-defined systematic consult triggers in place. Our study question was focused on patients experiencing death. Thus, while not a limitation in the study, our findings are not generalizable to all patients in the CVICU for whom PPC would be appropriate. In our center, PPC is limited to the inpatient setting, thus continuity with outpatient care was not available to maintain closer management of existing PPC patients. We were also not able to capture parental refusal of palliative care services if this occurred, which could be a point of exploration for future work.

Regrettably, while the history of a DNR discussion was documented, DNR status itself was inconsistently found in the medical record as reviewed above, and not in a standardized fashion, thus could not be analyzed. Although PPC documentation is often standardized, the exact date of DNR/DNI agreement was not included in PPC notes or readily ascertained from details in notes.

The explanation for why many PPC patients did not have a DNR discussion may be driven by the timing of PPC given that not all PPC patients had PPC proximal to significant changes in their prognosis or care, or, more likely, that DNR discussion was inconsistently documented in our EMR. This point itself highlights the limitation in conclusions about which patient’s experienced meaningful DNR discussions. As seen in Table 2, the separation of DNR discussion as “never”, “prior to DOD” and “initial on DOD” may highlight that while PPC involvement is strong, formal DNR discussion is not consistently documented, which may be point another quality improvement initiative at our institution.

## Conclusion

Most patients who died in our CVICU for whom PPC involvement was appropriate received timely consultation. However, many patients did not receive early PPC and most patients who did not receive PPC would have qualified under CAPC guidelines. High rates of PPC referral may have resulted from increased PPC awareness among intensivists, likely reflecting the increasing publications and interest. While most patients in this study who qualified for palliative care involvement did receive it, further investigation and education is needed particularly with cross-education between palliative care and CVICU teams, to more seamlessly integrate palliative care and standardize referrals. Further work to improve palliative care integration in the CVICU setting should involve PPC consults as a function of a checklist or automatic order set for admissions.

## Data Availability

No datasets were generated or analysed during the current study.
